# Dynamic LVOT Obstruction After Mitral Valve Repair in HCM: Treatment With a Cardiac Myosin Inhibitor

**DOI:** 10.1016/j.jaccas.2026.109225

**Published:** 2026-07-09

**Authors:** El-Moatasem Gabr, Aravinthan Vignarajah, Gautam Shah

**Affiliations:** a Department of Medicine, Cleveland Clinic Fairview Hospital, Cleveland, Ohio, USA; b Heart, Vascular, and Thoracic Institute, Cleveland Clinic, Cleveland, Ohio, USA

**Keywords:** cardiomyopathy, mitral valve, valve repair

## Abstract

**Background:**

Mitral valve repair (MVr) can alter left ventricular outflow tract (LVOT) geometry and precipitate dynamic LVOT obstruction (LVOTO) in patients with underlying hypertrophic cardiomyopathy (HCM). Cardiac myosin inhibitors are now a Class I recommendation for symptomatic obstructive HCM, yet their role in post-surgical LVOTO remains undefined.

**Case Summary:**

A 66-year-old woman with prior MVr developed progressive symptomatic dynamic LVOTO with resting and Valsalva gradients of 33 and 48 mmHg, systolic anterior motion (SAM), and NYHA class II–III symptoms. Mavacamten produced marked gradient reduction to 7 mmHg at rest and with Valsalva, with SAM resolution and symptomatic improvement to NYHA class II. Dose reduction to 2.5 mg led to gradient recurrence; re-escalation to 5 mg restored suppression, suggesting a reproducible dose-dependent effect.

**Discussion:**

Mavacamten effectively reduced dynamic LVOTO despite surgically altered mitral geometry, expanding the therapeutic scope of myosin inhibition to a post-surgical population not well represented in the major trials.

**Take-Home Messages:**

Dynamic LVOTO may emerge or progress after mitral valve repair in patients with underlying hypertrophic cardiomyopathy. Mavacamten may provide effective non-surgical suppression of post-repair dynamic LVOTO despite surgically altered mitral geometry.

## Visual Summary

**Figure:**
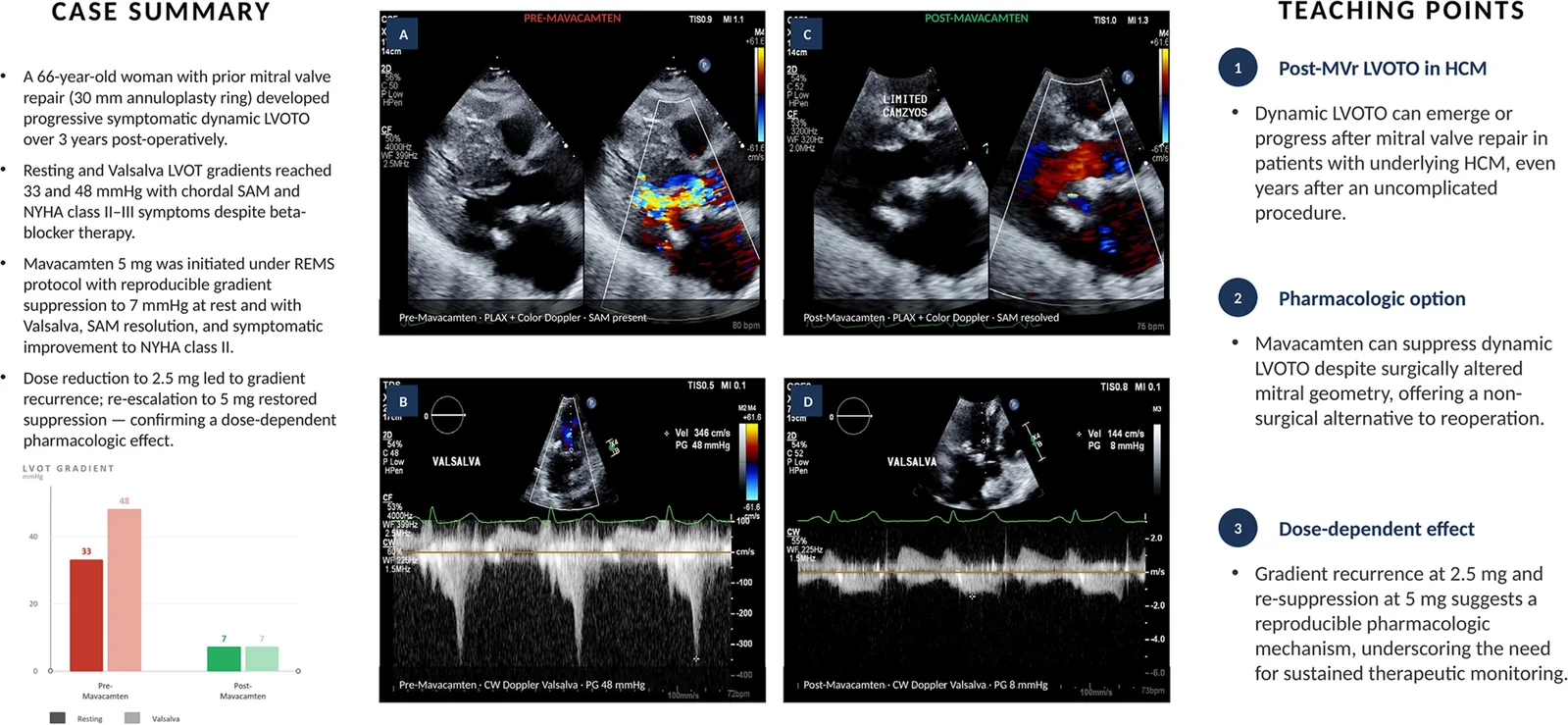

**Visual Summary:**
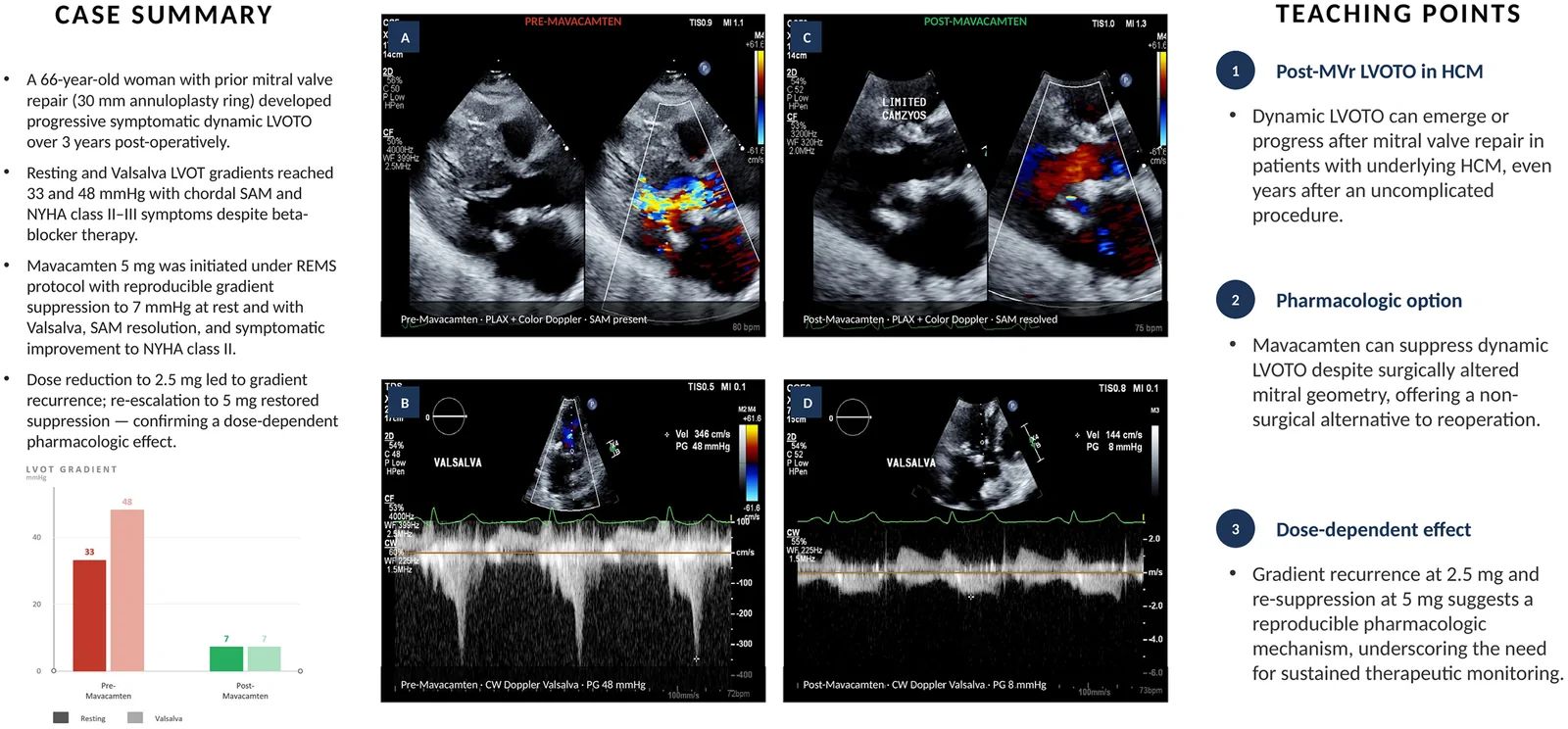
Mavacamten for Dynamic LVOT Obstruction After Mitral Valve Repair in HCM Case summary and key learning points of a patient with dynamic left ventricular outflow tract (LVOT) obstruction following mitral valve repair, treated with a cardiac myosin inhibitor, resulting in significant reduction in LVOT gradient and improvement in symptoms. CW = continuous wave; HCM = hypertrophic cardiomyopathy; LVOTO = left ventricular outflow tract obstruction; MVr = mitral valve repair; PG = peak gradient; PLAX = parasternal long-axis; REMS = Risk Evaluation and Mitigation Strategy; SAM = systolic anterior motion.

Obstructive hypertrophic cardiomyopathy (HCM) is among the most common inherited myocardial disorders, with a population prevalence of approximately 1 in 200 to 500 individuals.^1^ Its hallmark—dynamic left ventricular outflow tract obstruction (LVOTO)—results from an interplay between basal septal hypertrophy, a hyperdynamic underfilled left ventricle, and systolic anterior motion (SAM) of the mitral leaflets and subvalvular apparatus.^2^^,^^3^ The resulting obstruction is load-dependent, highly variable, and can be correlated mechanistically to excess myosin cross-bridge formation underlying the hypercontractile state in HCM.

**Take-Home Messages**

• Dynamic LVOTO may emerge or progress after mitral valve repair in patients with underlying hypertrophic cardiomyopathy.
• Mavacamten may provide effective nonsurgical suppression of postrepair dynamic LVOTO despite surgically altered mitral geometry.

Mitral valve repair (MVr) for degenerative mitral regurgitation introduces additional anatomic alterations through annuloplasty ring placement and leaflet modification, potentially altering mitral-aortic continuity, narrowing the aortomitral angle, and shifting the coaptation line anteriorly toward the septum. Reported rates of SAM-related LVOTO following MVr range from 2% to 16%, depending on patient anatomy and surgical technique.^4^^,^^5^ In susceptible patients, particularly those with preexisting septal hypertrophy, small left ventricular (LV) cavity, and residual redundant leaflet tissue, postoperative or delayed dynamic obstruction can be difficult to distinguish from structurally fixed outflow narrowing, with important implications for management.

Mavacamten is a first-in-class, selective allosteric inhibitor of cardiac myosin that shifts myosin heads toward a sequestered super-relaxed state, reducing the number of myosin heads available for actin cross-bridge formation.^6^ In the EXPLORER-HCM (Clinical Study to Evaluate Mavacamten [MYK-461] in Adults With Symptomatic Obstructive Hypertrophic Cardiomyopathy) and VALOR-HCM (A Study to Evaluate Mavacamten in Adults With Symptomatic Obstructive Hypertrophic Cardiomyopathy Who Are Eligible for Septal Reduction Therapy) trials, mavacamten produced significant and sustained reductions in resting and provoked left ventricular outflow tract (LVOT) gradients, improved NYHA functional class, and substantially reduced eligibility for invasive septal reduction therapy.^7^^,^^8^ However, both trials systematically excluded patients with prior cardiac surgery, leaving the drug's role in postsurgical LVOTO largely undefined.

We report a case of progressive, symptomatic dynamic LVOTO following MVr in a patient with underlying HCM, in whom mavacamten produced reproducible and sustained gradient suppression across multiple treatment cycles.

## History of presentation

A 66-year-old woman was referred for evaluation of progressive exertional dyspnea. Her cardiac history was notable for mitral valve repair for severe mitral regurgitation due to flail anterior mitral valve leaflet, with placement of a 30-mm Corcym Memo 4D annuloplasty ring and left atrial appendage ligation. Following uneventful recovery, she developed gradually worsening exertional dyspnea over the succeeding 2 years, with NYHA functional class II-III symptoms by 3 years post-MVr.

## Past medical history

The patient's medical history was notable for hypertension, chronic kidney disease (stage III), chronic obstructive pulmonary disease, and hepatitis C. Her cardiac surgical history included mitral valve repair and left atrial appendage ligation.

## Differential diagnosis

The differential for progressive dyspnea with an elevated LVOT gradient after MVr included dynamic LVOTO due to SAM in the setting of underlying HCM and surgically altered mitral-aortic geometry; fixed subaortic/valvular obstruction from residual or redundant chordal tissue; recurrent mitral regurgitation; and noncardiac dyspnea given her chronic obstructive pulmonary disease and chronic kidney disease. The marked lability of the gradient with Valsalva and the presence of chordal/mitral SAM favored a predominantly dynamic mechanism.

## Investigations

Preoperative echocardiography documented moderate interventricular septal hypertrophy (interventricular septum 1.5 cm), preserved LV systolic function (ejection fraction 60% to 65%), and a resting LVOT gradient of 11 mm Hg with a provoked gradient of 15 mm Hg. Serial postoperative echocardiography documented progressive dynamic LVOTO with chordal and mitral tissue SAM, moderate septal hypertrophy (interventricular septum up to 1.6 cm), and a persistently small LV cavity. A 0.6 × 0.4-cm echo-dense structure was identified on the ventricular aspect of the anterior mitral leaflet on multiple studies, likely consistent with residual or redundant chordal tissue. By 3 years post-MVr, resting and provoked gradients had risen to 25 mm Hg and 33 mm Hg, respectively. Beta-blocker therapy was in place but insufficient to bring gradients or symptoms under meaningful control.

## Management

Mavacamten was initiated in accordance with the standard Risk Evaluation and Mitigation Strategy protocol, with serial echocardiographic monitoring at scheduled intervals. Although metoprolol was prescribed before mavacamten initiation and increased early after mavacamten initiation, the subsequent recurrence of LVOTO after mavacamten dose reduction and repeat suppression following reescalation occurred on stable beta-blocker therapy, supporting a dose-dependent mavacamten effect. The echocardiographic timeline is presented in Table 1. Within 4 weeks of initiation, resting and provoked gradients fell to 5 mm Hg and 20 mm Hg, respectively.

**Table 1:** Serial Echocardiographic Data

Date	IVS Length, Leaflet Tips, cm	SAM	Rest LVOT, mm Hg	Valsalva, mm Hg	HR beats/min	EF, %	Metoprolol Dose	Key Observations
Pre-MVr	1.3	Chordal	11	15	69	75	None	Baseline; moderate septal LVH; clinically quiescent
3 years post MVr	1.6	Chordal	25	33	82	70	50 mg XL	Progressive LVOTO; started on mavacamten 5 mg daily
1st REMS 4-week ECHO	1.6	Chordal	5	20	76	70	50 mg XL	Continue mavacamten 5 mg daily, Increase metoprolol to 100 mg XL daily
2nd REMS 8-week ECHO	1.4	Chordal	5	18	81	68	100 mg XL	Decreased mavacamten dose to 2.5 mg daily per REMS protocol
3rd REMS 12-week ECHO	1.6	Mild	26	33	79	55	100 mg XL	Gradient recurrence. Increased mavacamten dose to 5 mg
1st REMS dose-change 4-week ECHO	1.6	Mild	5	7	76	58	100 mg XL	Optimal response after adherence reestablished
REMS 3-month ECHO	1.5	Chordal	33	48	78	59	100 mg XL	Possible drug to drug interaction/medication adherence variability from a UTI; continue mavacamten 5 mg daily
REMS 6-month ECHO	1.2	None	7	7	72	55	100 mg XL	Continue mavacamten 5 mg

ECHO = echocardiogram; EF = left ventricular ejection fraction; HR = heart rate in beats per minute; IVS = interventricular septum; LVH = left ventricular hypertrophy; LVOT = left ventricular outflow tract; LVOTO = left ventricular outflow tract obstruction; MVr = mitral valve repair; REMS = Risk Evaluation and Mitigation Strategy; SAM = systolic anterior motion; UTI = urinary tract infection.

## Outcome and follow-up

**Figure 1:**
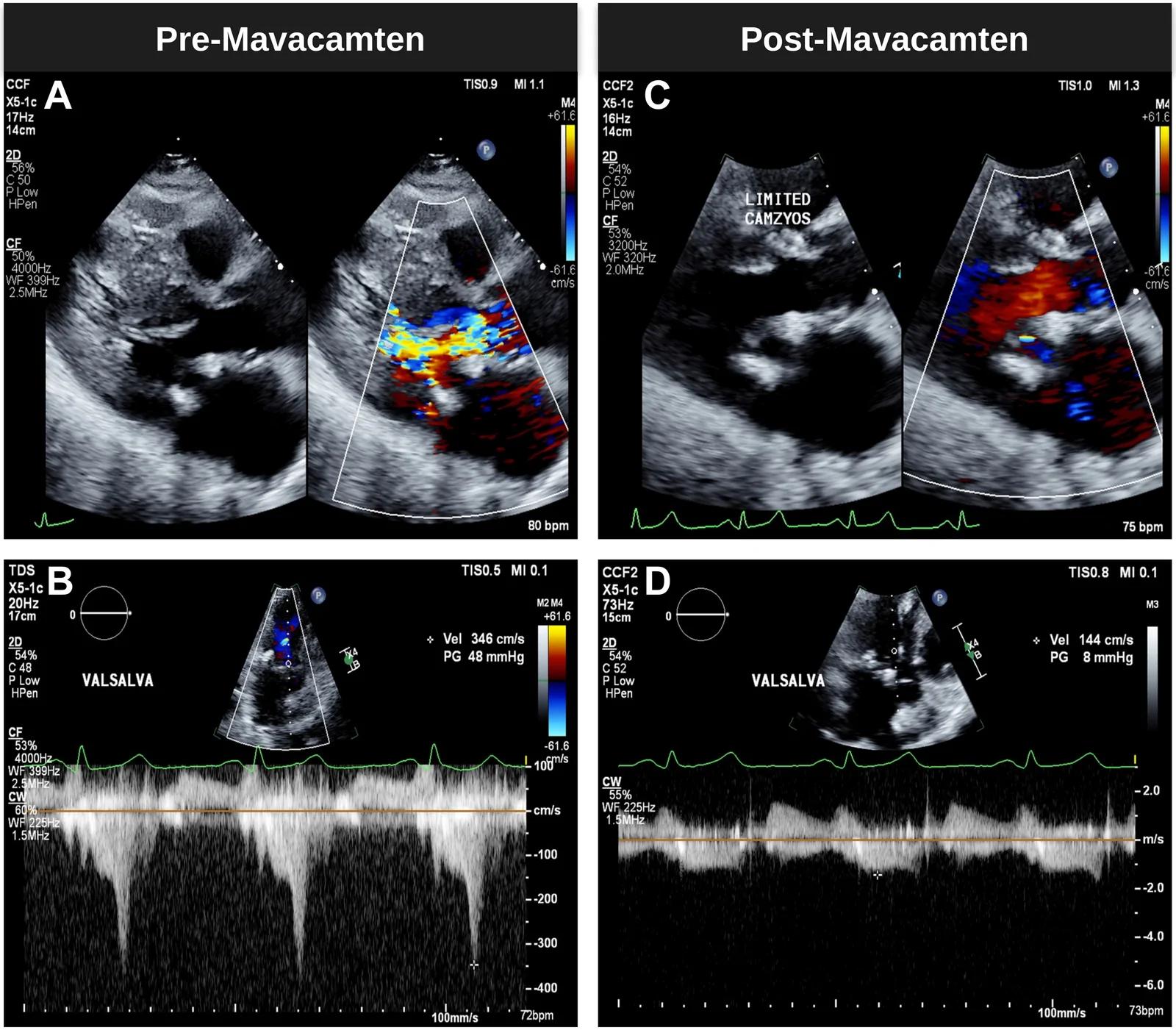
Echocardiographic Assessment Before and After Mavacamten Therapy (A and B) Pretreatment. (A) Parasternal long-axis (PLAX) view with and without color Doppler demonstrating chordal systolic anterior motion (SAM) and left ventricular outflow tract (LVOT) flow acceleration. (B) Continuous-wave (CW) Doppler across the LVOT following Valsalva maneuver, demonstrating an elevated peak gradient (PG 48 mm Hg). (C and D) Post-mavacamten treatment. (C) PLAX view with and without color Doppler showing resolution of chordal SAM and reduction in LVOT obstruction. (D) CW Doppler across the LVOT following Valsalva maneuver demonstrating marked reduction in peak gradient after mavacamten therapy (PG 8 mm Hg).

At 8 weeks, mavacamten dose was decreased to 2.5 mg daily per Risk Evaluation and Mitigation Strategy protocol; by the 12-week echocardiogram, gradients had recurred (rest 26 mm Hg, Valsalva 33 mm Hg) with mild SAM, prompting reescalation to 5 mg daily. Following reescalation and a period of confirmed adherence after earlier possible noncompliance, the patient achieved an optimal response: resting gradient of 5 mm Hg, Valsalva gradient of 7 mm Hg, and complete resolution of SAM. Symptoms improved markedly, with greater ability to perform activities of daily living, including climbing 4 flights of stairs in 2-flight increments, consistent with improvement to NYHA functional class II. Representative echocardiographic images before and after mavacamten therapy are shown in Figure 1.

Approximately 3 months later, gradients again returned to baseline levels (rest 33 mm Hg, Valsalva 48 mm Hg) with recurrence of mild SAM, possibly related to medication adherence variability during a urinary tract infection, providing the functional equivalent of a second pharmacologic rechallenge. With continued mavacamten 5 mg daily, gradients fell to 7 mm Hg at rest and with Valsalva at the 6-month follow-up, with absent SAM, sustained symptomatic improvement, and LV ejection fraction preserved at 55%, with no documented episodes below 50% throughout.

## Discussion

Dynamic LVOTO in HCM classically reflects the convergence of 3 contributors: basal septal hypertrophy, a hypercontractile and geometrically constrained LV, and SAM of the mitral apparatus.^2^^,^^3^ Surgical repair superimposes additional complexity. The annuloplasty ring contracts the posterior annulus, shifts the coaptation point anteriorly, and narrows the aortomitral angle, collectively reducing clearance between the mitral apparatus and the septum during systole.^4^^,^^5^ Residual or redundant leaflet tissue, such as the echo-dense structure observed at the anterior leaflet in this patient, further encroaches on the LVOT and amplifies drag-mediated anterior displacement. Despite this structural complexity, the marked reversibility of gradients and recurrence of obstruction following dose reduction support dynamic LVOTO as the principal driver of obstruction.

EXPLORER-HCM and VALOR-HCM established mavacamten efficacy in obstructive HCM, reducing LVOT gradients, improving functional class, and deferring septal reduction therapy, leading to a Class I recommendation in the 2024 American Heart Association/American College of Cardiology guidelines.^7^^,^^8^^,^^9^ Neither trial, however, enrolled patients with prior MVr. This case suggests the pharmacologic rationale remains constant in surgically altered patients, provided the obstruction is predominantly dynamic.

This case highlights 3 practical considerations. First, worsening or progression of dynamic LVOTO after MVr is possible in patients with dynamic physiology. Second, such LVOTO obstruction after MVr can respond to mavacamten and avoid reoperation in a previously repaired field. Third, the dose-dependent gradient response observed in this case, recurrence of dynamic LVOTO with dose reduction to 2.5 mg and repeat resolution of LVOTO on reescalation to 5 mg, suggested that sustained therapeutic dosing may be required for maintained gradient control. Although concomitant beta-blocker therapy may have contributed to gradient reduction, the reproducible recurrence and suppression of LVOTO following mavacamten dose adjustment while background therapy remained stable supports a clinically meaningful pharmacologic effect.

## Conclusions

This case demonstrates that mavacamten can produce reproducible, sustained reduction of dynamic LVOTO in a patient with prior mitral valve repair, despite the structural complexity introduced by annuloplasty. The reproducible pattern of suppression on therapy, recurrence with dose deescalation, and resuppression following reescalation supports dynamic hypercontractility as the dominant modifiable driver of obstruction.

## Funding Support and Author Disclosures

The authors have reported that they have no relationships relevant to the contents of this paper to disclose.
